# Development of a phenotypic screening assay to measure activation of cancer-associated fibroblasts

**DOI:** 10.3389/fphar.2025.1526495

**Published:** 2025-02-13

**Authors:** Marcus M. Ilg, Alice R. Lapthorn, Sophie L. Harding, Tariq Minhas, Gouri Koduri, Stephen A. Bustin, Selim Cellek

**Affiliations:** ^1^ Fibrosis Research Group, Medical Technology Research Centre, Anglia Ruskin University, Chelmsford, United Kingdom; ^2^ The Essex Cardiothoracic Centre, Basildon University Hospital, Basildon, United Kingdom; ^3^ Southend University Hospital NHS Foundation Trust, Westcliff-on-Sea, United Kingdom

**Keywords:** phenotypic, cancer, metastasis, fibroblast, myofibroblast, screening, assay, drug discovery

## Abstract

**Background:**

In cancer metastasis, tumor cells condition distant tissues to create a supportive environment, or metastatic niche, by driving the activation of cancer-associated fibroblasts (CAFs). These CAFs remodel the extracellular matrix, creating a microenvironment that supports tumor growth and compromises immune cell function, enabling cancer cells to evade immune detection. Consequently, targeting the activation of CAFs has been proposed as a therapeutic strategy to hinder metastatic spread. Our objective was to develop the first *in vitro* phenotypic screening assay capable of assessing this activation process.

**Methods:**

Human primary lung fibroblasts were co-cultured with highly invasive breast cancer cells (MDA-MB-231) to identify changes in the expression of selected genes using RT-qPCR. An In-Cell ELISA (ICE)-based assay using human lung fibroblasts, MDA-MB-231 cells and human monocytes (THP-1 cells) was developed to measure the activation of CAFs. Another ELISA assay was used to measure released osteopontin.

**Results:**

When lung fibroblast were co-cultured with MDA-MB-231 cells, among the 10 selected genes, the genes for osteopontin (SPP1), insulin like growth factor 1 (IGF1), periostin (POSTN) and α-smooth muscle actin (α-SMA, ACTA2) elicited the greatest fold change (55-, 37-, 8- and 5-fold respectively). Since osteopontin, IGF-1 and periostin are secreted proteins and α-SMA is an intracellular cytoskeleton protein, α-SMA was chosen to be the readout biomarker for the ICE assay. When fibroblasts were co-cultured with MDA-MB-231 cells and monocytes in the 96 well ICE assay, α-SMA expression was increased 2.3-fold yielding a robust Z′ of 0.56. A secondary, low throughput assay was developed by measuring the release of osteopontin which showed a 6-fold increase when fibroblasts were co-cultured with MDA-MB-231 cells and monocytes.

**Discussion:**

This phenotypic assay is the first to measure the activation of CAFs in a 96-well format, making it suitable for medium-to high-throughput screening of potential therapeutic compounds. By focusing on observable cellular phenotypic changes rather than targeting specific molecular pathways, this assay allows for a broader and unbiased identification of compounds capable of modulating CAF activation.

## 1 Introduction

Metastasis accounts for 90% of cancer-related deaths, a statistic that has seen little improvement over the past 50 years (Faguet, 2008). In 2020 alone, nearly 10 million cancer deaths were recorded globally (Sung et al., 2021), highlighting the urgent need for treatments that prevent tumor spread. The transition from primary tumor to distant metastatic colonization depends on multiple factors, including interactions with local and distant microenvironments (Gupta and Massagué, 2006). A key component is the extracellular matrix (ECM), which supports cancer progression by promoting cell survival, migration, and metastasis formation (Frantz et al., 2010; Rozario and DeSimone, 2010; Mouw et al., 2014). Cancer progression involves dysregulation of ECM dynamics, which allows invading cancer cells to survive, colonize, and expand to form macro-metastases.

Despite interest in targeting the ECM therapeutically (Lu et al., 2012), degrading the ECM within the primary tumor has often led to unwanted cancer cell dissemination (Fang et al., 2014). A safer approach focuses on targeting cells that remodel the ECM within the metastatic niche. Tumor cells can “corrupt” resident fibroblasts in distant tissue by inducing their transformation/activation into cancer-associated fibroblasts (CAFs) that secrete ECM proteins to support metastatic growth (Joyce and Pollard, 2009; Psaila and Lyden, 2009; Peinado et al., 2017). The initiation of metastatic growth at a distant site has been described as a major bottle neck in cancer progression, lending itself as an ideal therapeutic window; right after surgery and combined with adjuvant treatment (Celià-Terrassa and Kang, 2016).

The origin of CAFs can be tissue resident fibroblasts, adipocytes, pericytes, endothelial cells or bone marrow-derived mesenchymal stem cells (MSCs) (Sahai et al., 2020). CAFs can be activated within the cancer microenvironment by physical changes to ECM (stiffness and composition), cell-to-cell contact signals (such as Notch), DNA damage (chemotherapy or radiotherapy), physiological stress (i.e., disrupted metabolism), inflammatory signals (e.g., transforming growth factor-β [TGFβ], interleukins 1 and 6 [IL-1, IL-6], tumor necrosis factor [TNF]) and growth factors (e.g., platelet derived growth factor [PDGF] and fibroblast growth factor [FGF]) (Sahai et al., 2020). Once activated, CAFs adopt a high ECM-producing and remodeling phenotype similar to myofibroblasts in fibrosis and produce high levels of TGFβ and α-smooth muscle actin (α-SMA) (Tomasek et al., 2002). Activated CAFs are then able to promote cancer invasion by ECM remodeling (Sahai et al., 2020) and play important role in tumor immune microenvironment (TIME) (Mao et al., 2021; Wu et al., 2023).

Monocytes and macrophages have been long implicated as major regulators of cancer metastasis. Molecular cross-talk between CAFs and monocytes/macrophages has been shown to be bi-directional: CAFs can recruit monocyte/macrophages and induce their activation (Cho et al., 2018; Du et al., 2020; Jia et al., 2023) while monocytes/macrophages when activated by CAFs can lose their tumoricidal abilities and can suppress T cell proliferation which can lead to cancer cells evading immune detection (Pakravan et al., 2022).

Breast carcinoma is the most common cancer among women worldwide and about 20%–30% of patients develop metastatic disease when diagnosed with early breast cancer. Whilst breast cancer can spread to bone, lung, liver and brain, it has been reported that 60%–70% of breast cancer patients who eventually died were diagnosed with lung metastasis (Jin et al., 2018; Chen et al., 2019). Our goal was to create the first phenotypic screening assay capable of measuring this activation of CAFs in response to interactions with breast cancer cells and immune cells. Using co-cultures of primary human lung fibroblasts, breast cancer cells, and monocytes, we aimed to replicate the lung microenvironment encountered by disseminated breast cancer cells. This assay offers a novel, unbiased tool for discovering adjuvants that can disrupt metastatic niche formation, with the potential to improve outcomes when combined with standard chemotherapy following tumor resection.

## 2 Materials and methods

### 2.1 Ethics for lung fibroblasts

Healthy lung tissue was obtained from patients undergoing lung resection surgery at The Essex Cardiothoracic Centre, University Hospital Basildon and Thurrock, Essex, United Kingdom. The patients were fully informed and gave written consent to the study. The study was approved by London–Surrey NHS Research Ethics Committee (22/PR/0499) and Health Research Authority (IRAS: 314940). The cells used in this study were isolated from two patient samples: one patient (age 70; co-morbidity COPD) was operated on for adenocarcinoma on the upper lobe, the other patient (age 70; co-morbidities bowel cancer and hypertension) was operated on for atrial myxoma. The lung tissue used in this study were resected from non-cancerous areas.

### 2.2 Cell culture

Primary human lung fibroblasts were isolated via explant technique as previously described (Ilg et al., 2019; Ilg et al., 2022; Lapthorn et al., 2022; Lapthorn et al., 2024). Tissue samples (3 × 3 mm) were anchored to the bottom of a 6-well plate (NUNC, Fisher Scientific, United Kingdom) in DMEM-F12 (GIBCO, Invitrogen, United Kingdom) containing 10% FCS (Fisher Scientific, United Kingdom) and 1% penicillin-streptomycin (GIBCO, Invitrogen, United Kingdom) at 37°C, 5% CO_2_ for 5–7 days. Tissue fragments were carefully removed using forceps when cell outgrowth was observed after 7 days. The cells were transferred to T75 cell culture flasks (Fisher Scientific, United Kingdom) when confluent and were then further expanded with simultaneous preparation of stocks. Passages 2–5 were used for all experiments presented in this work in order to avoid spontaneous transformation/activation (Baranyi et al., 2019).

MDA-MB-231 cells were obtained from American Type Culture Collection (ATCC) and cultured in DMEM-F12 (GIBCO, Invitrogen, United Kingdom) containing 10% FCS (Fisher Scientific, United Kingdom) and 1% penicillin-streptomycin (GIBCO, Invitrogen, United Kingdom) at 37°C, 5% CO_2_. THP-1 cells were obtained from ATCC and cultured in RPMI (GIBCO, Invitrogen, United Kingdom) containing 10% FCS (Fisher Scientific, United Kingdom) at 37°C, 5% CO_2_.

### 2.3 Immunocytochemistry

Primary human lung fibroblasts were seeded onto glass coverslips (Fisher Scientific, United Kingdom) in 6-well plates at a density of 5 × 10^4^ cells per well, and left to adhere overnight at 37°C, 5% CO_2_. Cells were incubated with or without 10 ng/mL TGF-β1 (Sigma-Aldrich, United Kingdom) for 72 h. Cells were fixed in ice cold methanol for 10 s and the coverslips washed three times in PBS, before blocking using 10% donkey serum (Sigma-Aldrich, United Kingdom) in PBS for 1 h at room temperature in a humidified chamber. The coverslips were incubated with the primary antibodies diluted to their desired concentration in PBS, (anti-vimentin, 1:1,000, Abcam, United Kingdom; anti-desmin, 1:500, Abcam, United Kingdom; anti-α-SMA, 1:1,000) for 2 h at room temperature in a humidified chamber. Coverslips were washed three times with PBS, before incubation with the secondary antibody diluted in PBS (anti-mouse AlexaFlour^®^ 488, 1:500, Abcam, United Kingdom) for 2 h at room temperature in a humidified chamber. The coverslips were washed again, before mounting onto slides using mounting medium with DAPI (Vector Laboratories, United States).

### 2.4 RNA isolation

Fibroblasts were seeded into clear 6-well plates at a density of 8 × 10^4^ cells/well. After overnight attachment, 4 × 10^4^ cells/mL of MDA-MB-231 cells were added and cells were incubated for 3, 5, or 7 days before RNA was extracted from cells using the RNeasy Mini Kit (Qiagen, United Kingdom), according to the manufacturer’s instructions, after which RNA was suspended in 40 µL of RNase-free water and stored at −80°C.

### 2.5 Primers and probes

All assays were designed using the Beacon Designer 8.21 qPCR assay design software package (Premier Biosoft, San Francisco, CA, United States). Sequences specifying the target genes were downloaded from the NIH National Centre for Biotechnology Information website. The specificity of primers and amplicons were analyzed *in silico* using Primer-BLAST (https://www.ncbi.nlm.nih.gov/tools/primer-blast/) and BLAST (https://blast.ncbi.nlm.nih.gov/Blast.cgi). All oligonucleotides were synthesized and lyophilized by Sigma Aldrich (Haverhill, United Kingdom) and upon receipt resuspended in sterile RNase-free water at 100 µM and stored in aliquots at −20°C. Table 1 lists the details of the primers, amplicon length and PCR efficiency obtained using target-specific dilution curves.

**TABLE 1:** Details of primers, amplicon length, and PCR efficiency.

Target	Symbol	NCBI Reference	Forward primer (5′-3′)	Reverse primer (5′-3′)	Amplicon (bp)	Efficiency (%)
Cyclin Dependent Kinase Inhibitor 1A	CDKN1A	NM_000389.5	CTG​GAG​ACT​CTC​AGG​GTC​GAA	GGA​TTA​GGG​CTT​CCT​CTT​GGA	98	97%
Periostin	POSTN	NM_006475.3	GAA​AGG​GAT​CCT​TCA​CTT​AC	CTC​CAA​ACC​TCT​ACG​GAT​ATC	80	99%
Tenascin-C	TNC	NM_002160.4	AGAAGACTGCTCAGAGGT	CGCATCTCATTGTCCCAG	93	95%
Glyceraldehyde-3-Phosphate Dehydrogenase	GAPDH	NM_002046.7	AGCCACATCGCTCAGACA	TGACCAGGCGCCCAATAC	75	100%
Smooth muscle alpha actin-2	ACTA-2	NM_001141945.3	CTA​TGC​CTC​TGG​ACG​CAC​AAC	GAC​ATT​GTG​GGT​GAC​ACC​ATC​TC	64	99%
Secreted Phosphoprotein 1	SPP1	NM_001040058.2	GCT​GAT​TCT​GGA​AGT​TCT​GAG​GAA	TGG​GTC​AGG​GTT​TAG​CCA​TG	81	98%
Fibronectin	FN1	NM_212482.4	CCA​AGT​ACA​TTC​TCA​GGT​GG	GAT​GGT​GTA​GGA​GTT​TAA​GTG​G	89	93%
Transforming Growth Factor ß receptor	TGFBR3	NM_003243.5	TCG​GTC​TCC​AGT​TGG​TTA​TCT​G	GCA​AGA​GAA​GTA​AGG​CAA​TCC​AA	99	98%
SMAD family member 4	SMAD4	NM_005359.6	TTG​GGT​CAA​CTC​TCC​AAT​G	TGCACACCTTTGCCTATG	74	100%
Insulin-like growth factor 1	IGF-1	NM_001111283.3	GTG​CTG​CTT​TTG​TGA​TTT​CTT​GA	CAG​AGC​TGG​TGA​AGG​TGA​G	98	99%
Thrombospondin	THBS1	NM_003246.4	CAC​AGA​TGT​TGA​TGA​GTG​CAA​AG	GGTCCGTGTTCTCACACC	80	101%
RB Transcriptional Co-repressor like 1	RBL1	NM_002895.4	ATT​TGG​CAT​GGA​AAC​CAG​AG	GTC​ACC​CTT​CTG​GGA​GTC​AA	154	94%
3-Hydroxy-3-Methylglutaryl-CoA Reductase	HMGCR	NM_000859.3	TAC​AGA​TAC​TTG​GGA​ATG​CAG​AG	CTT​GTA​GGC​TGG​GAT​ATG​CTT​A	96	102%

### 2.6 cDNA synthesis

Fibroblast RNA samples were reverse transcribed with SuperScript IV (ThermoFisher, Waltham, MA, United States). All reagents were kept on ice prior to carrying out the reverse transcription step in 20 µL using 100U RT, 100 ng random primers and 0.2 mM of each dNTP. Reaction conditions were 25°C for 5 min, 55°C for 5 min and 85°C for 5 min cDNA samples were diluted with equal volumes (20 µL) of nuclease-free water (ThermoFisher, United Kingdom) and stored at −20°C.

### 2.7 qPCR reactions

qPCR assays were carried out using SensiFast master mix (Bioline, London, United Kingdom) and 0.5 µL of cDNA per 10 µL qPCR reaction volume containing primers at final concentrations of 500 µM and, where appropriate, a probe at 250 µM final concentration. PCR conditions were 1 min at 95°C, followed by 40 cycles of 1 s each at 95°C and 60°C. Assays were carried out either in heat-sealed white qPCR 96 well plates on BioRad CFX Connect and CFX Opus qPCR instruments (BioRad, Watford, United Kingdom) or adhesive-sealed well plates on a Techne PrimePro 48 cycler (Cole-Palmer, St. Neots, United Kingdom).

### 2.8 qPCR data analysis

All qPCR data were initially analysed using the software provided with each instrument, then exported for further analysis in Microsoft Excel for Mac (v.16.80) and PRISM for Mac (v.10). Dilution curves were used to calculate the PCR efficiencies of the various assays. ∆Cq values from replicate experiments were aggregated, and ∆∆Cq values were computed relative to the geometric mean of three reference genes. These reference genes were selected based on their minimal variability across the 7-day time course. ∆Cq values are shown with ±95% Confidence Intervals (CI). The 2-fold change was chosen as a cut-off for significant change; based on our previous observation that relative mRNA expression levels vary between 2- and 3-fold, depending on the efficiency of the RT step (Bustin et al., 2015).

### 2.9 In-cell ELISA

An In-Cell ELISA (ICE) assay to quantify α-SMA was adapted based on previously described protocols (Mateus et al., 2018; Ilg et al., 2019; 2020). Briefly, fibroblasts were seeded into 96-well, optical, flat-bottom, black microplates (NUNC, Fisher Scientific) at 4 × 10^3^ cells/well. After overnight attachment, they were incubated with or without 2 × 10^3^ cells/well of MDA-MB-231 and/or 2 × 10^3^ THP-1 cells/mL in 50% DMEM and 50% RPMI containing media (10% FCS, 1% penicillin-streptomycin) for 7 days. After the incubation, the cells were fixed with 4% paraformaldehyde, washed with phosphate-buffered saline (PBS) containing 0.1% Triton X-100 and blocked with 10% donkey serum in PBS containing 0.1% Triton X-100. This was followed by primary antibody incubation using an anti–α-SMA antibody (1:3,000; Sigma-Aldrich, United Kingdom) in PBS containing 0.1% Triton X-100 for 2 h. After washing steps, the cells were incubated with donkey anti-mouse secondary antibody conjugated to an infrared dye that emits at 800 nm (1:500; IRdye 800CW; Li-COR, Cambridge, United Kingdom) and a nuclear counterstain that emits at 700 nm (1:1,000; DRAQ5; Biostatus, United Kingdom) for 1 h. The plate was scanned using an infrared imaging system (Odyssey DLx imager; LI-COR) at both 700 and 800 nm wavelengths. No staining for α-SMA was measurable when no cells were used or when primary antibody was omitted.

### 2.10 Osteopontin ELISA

Human osteopontin ELISA Kit (Abcam, ab269374) was used according to the manufacturer’s instructions to quantify osteopontin in the conditioned media derived from fibroblasts in co-culture with other cells. The ELISA uses a mouse monoclonal antibody specific to human OPN (epitope SVVYGLR) which is the integrin-biding sequence and conserved in all human osteopontin isoforms (Yokosaki et al., 1999). Fibroblasts were seeded into clear 6-well plates at a density of 8 × 10^4^ cells/well. After overnight attachment, 4 × 10^4^ MDA-MB-231 and THP-1 cells/mL were added and were incubated for 3, 5, or 7 days before conditioned media was collected. Conditioned media was centrifuged at 2,000 g for 10 min to remove cell debris. A Bio-Rad plate reader was used to measure OD at 450 nm as an endpoint reading. A standard curve was generated to calculate osteopontin concentrations. R^2^ of the standard curve was above 0.9.

### 2.11 Statistical analysis

Experiments were performed in at least three technical repeats (N = 3) and using primary cells from two different patients. For analysis of two groups, Student’s t-test was used. For statistical analysis between multiple groups, one-way analysis of variance (ANOVA) was used. Data expressed as mean ± SD. A P value of less than 0.05 was considered statistically significant.

Robust Z-factor (RZ′) was used to confirm that the ICE assay was suitable for high-throughput screening. RZ′ is a variation of the Z′ factor and uses median and median absolute deviation (MAD) instead of mean and standard deviation. RZ′ is less sensitive to outliers than Z′. RZ′ above 0.5 proves that an assay is robust and reproducible for high-throughput screening (Zhang et al., 1999; Atmaramani et al., 2020).

The following formula was used to calculate RZ′:

$$RZ^{\prime }=1-\frac{3-\left(\text{MADp}+\text{MADn}\right)}{\left(\text{Mp}-\text{Mn}\right)}$$

Where p refers to the positive controls (fibroblasts + cancer cells + monocytes) and n refers to the negative controls (fibroblasts only). MADp and MADn are the median average deviations of the positive and negative controls, and Mp and Mn are the medians of the positive and negative controls.

## 3 Results

### 3.1 Validation of fibroblasts

**FIGURE 1:**
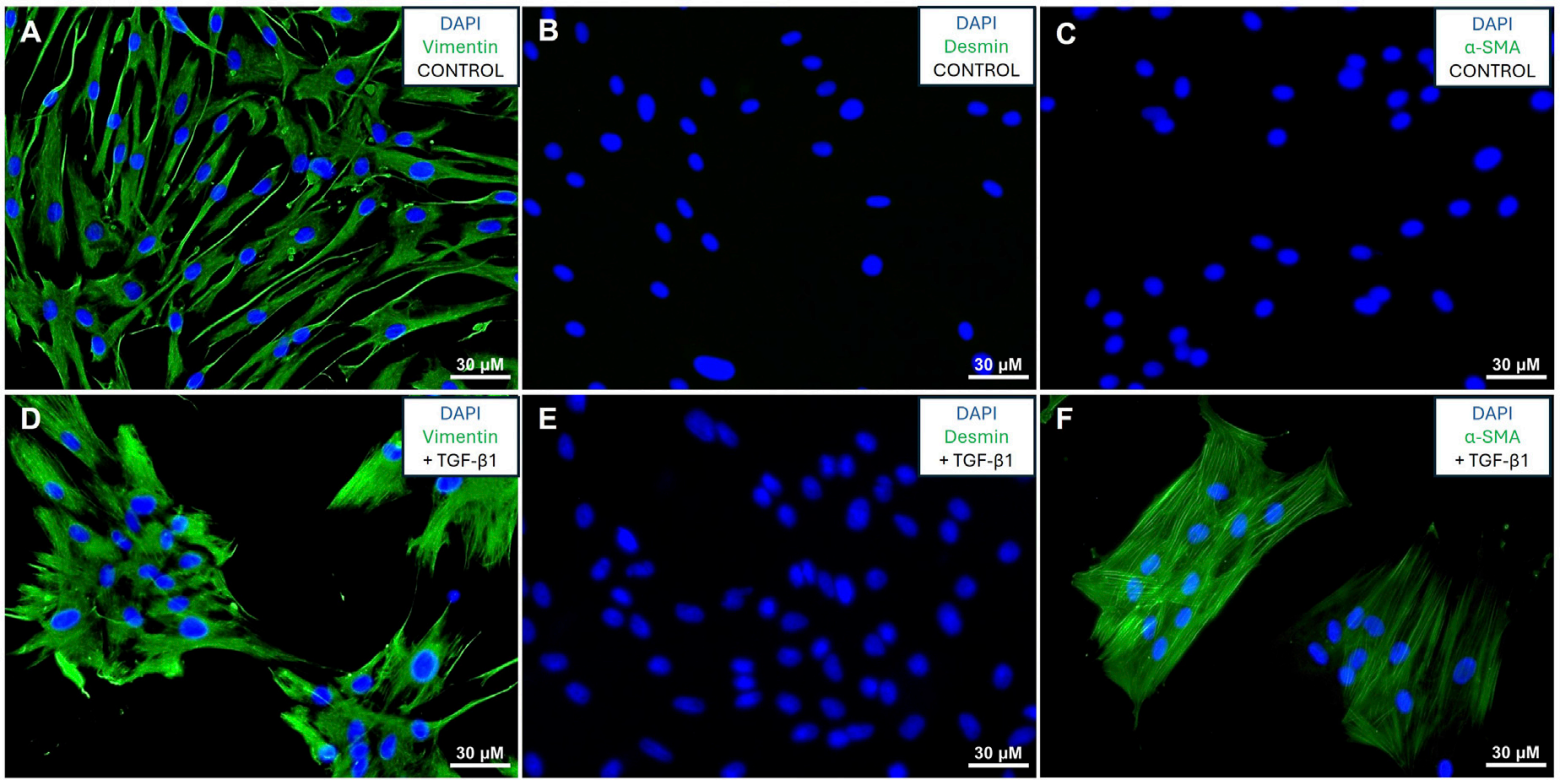
Validation of fibroblasts: Immunofluorescence staining of fibroblasts in the absence **(A–C)** or presence **(D–F)** of TGF-β1. Cells stained for vimentin **(A, D)**, desmin **(B, E)**, and α-SMA **(C, F)** with green signal denoting protein expression and blue signal showing DAPI stained nuclei.

The cells were isolated from fresh human lung tissue and propagated. Cells were characterized by immunofluorescence staining for vimentin, desmin and α-SMA in the presence and absence of TGF-β1 as previously described (Mateus et al., 2018; Ilg et al., 2022; Lapthorn et al., 2022; Lapthorn et al., 2024). The cells expressed the mesenchymal marker vimentin both in the presence and absence of TGF-β1 (Figures 1A, D), whilst not expressing any desmin (Figures 1B, E), and only expressing α-SMA in response to TGF-β1 treatment (Figures 1C, F), confirming the fibroblast identity of the isolated cells. The *de novo* expression of α-SMA in response to TGF-β1 confirms that these cells can be activated to myofibroblasts which represents MyoCAF phenotype (Kalluri, 2016).

### 3.2 Selection of biomarkers for assay development

**FIGURE 2:**
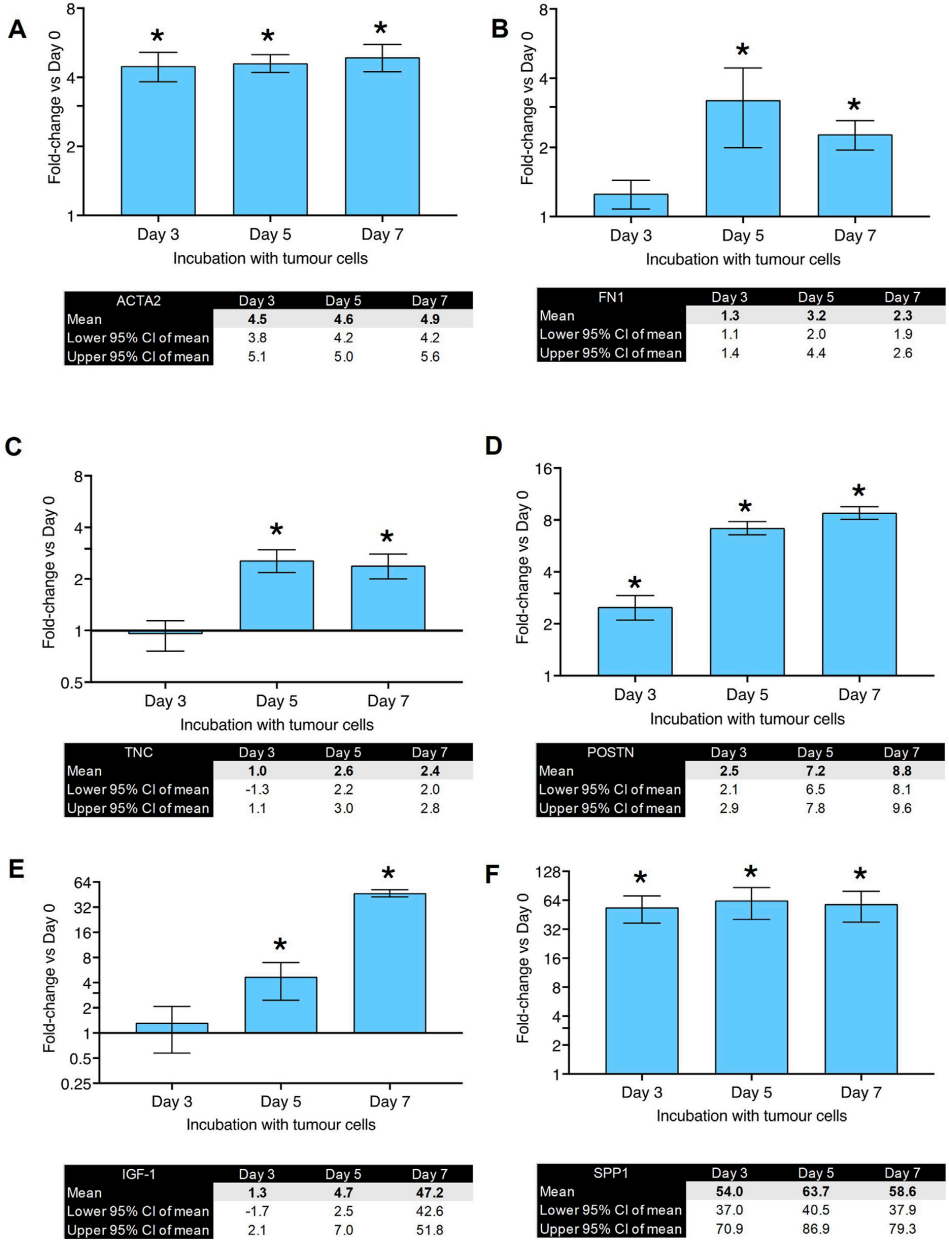
Gene expression changes in co-culture of human primary lung fibroblasts with MDA-MB-231 cells after various time points (part 1): Fold changes (±CI) compared to day 0 for **(A)** ACTA2, **(B)** FN1, **(C)** TNC, **(D)** POSTN, **(E)** IGF-1, **(F)** SPP1 measured using qPCR. Gene expression was normalized to three reference genes. The data were obtained from three replicates from two donors. * denotes minimum 2 fold change. Cq values are listed in Supplementary Table S1.

**FIGURE 3:**
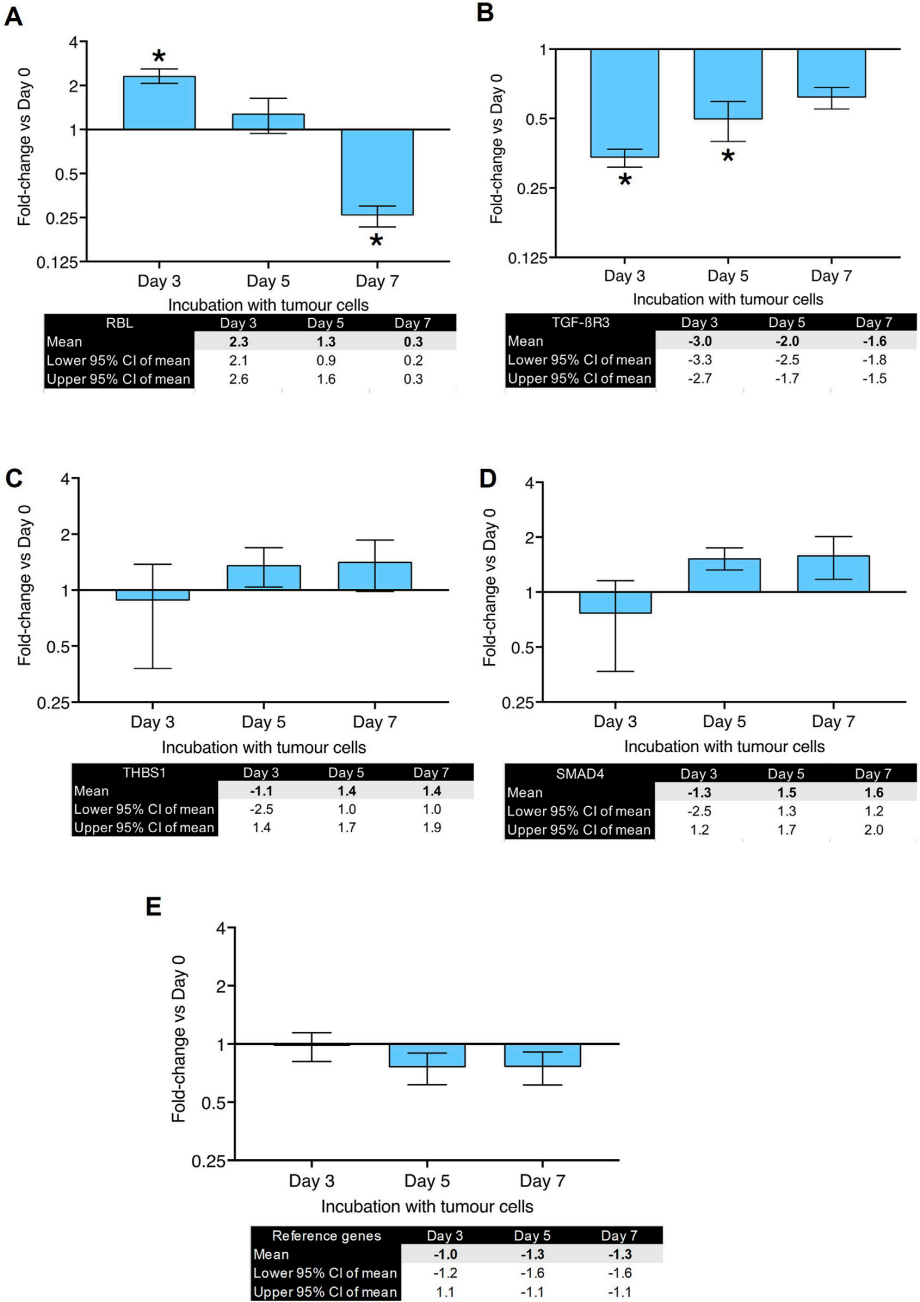
Gene expression changes in co-culture of human primary lung fibroblasts with MDA-MB-231 cells after various time points (part 2): Fold changes (±CI) compared to day 0 for **(A)** RBL, **(B)** TGFBR3, **(C)** THBS1, **(D)** SMAD4 measured using qPCR. Gene expression was normalized to three reference genes. Reference gene means are shown in panel **(E)**. The data were obtained from three replicates from two donors. * denotes minimum 2 fold change. Cq values are listed in Supplementary Table S1.

To investigate which biomarkers will show the most significant changes, primary human lung fibroblasts were co-cultured with MDA-MB-231 cells for 3, 5 and 7 days before RNA isolation. The change in expression was investigated in ten selected genes which have previously been reported to be involved in CAF differentiation (Louault et al., 2020). The results of the RT-qPCR experiments can be seen in Figures 2, 3. The fold-changes (±95% CI) are shown relative to expression levels in fibroblasts only. The Cq values are presented in Supplementary Table S1.

Figure 2A shows fold-changes in expression of ACTA2, which encodes the α-SMA protein and is characteristic of myofibroblast differentiation. Its expression peaked after 5 days of co-culture and showed a 5-fold-change. FN1 (gene for fibronectin, Figure 2B) and TNC (gene for tenascin C, Figure 2C) showed a peak of 3-fold increase on day 5. Figure 2D shows the changes in POSTN (gene for periostin) expression which peaked after 7 days with a fold-change of 8. IGF-1 (Figure 2E) showed a significant upregulation (37-fold-change) after 7 days of co-culture. SPP1, the gene for osteopontin, showed the highest fold-change (55-fold) observed after 5 days of co-culture (Figure 2F). RBL (gene for Retinoblastoma-Like Protein 1) was upregulated after 3 days of co-culture but downregulated after 7 days (Figure 3A). TGFBR3 (gene for Transforming Growth Factor Beta Receptor 3, Figure 3B) was downregulated at days 3 and 5. THSB1 (gene for thrombospondin 1, Figure 3C) remained largely unchanged in co-culture. Similarly, the gene for SMAD4 (Figure 3D) showed no significant changes in expression at any time point. Figure 3E depicts the reference genes used for these experiments and shows that these remained stable throughout the co-culture experiments, making them valid to be used for normalization of gene expression, in accordance with the MIQE guidelines (Bustin et al., 2009).

These experiments suggest that the co-culture of primary human lung fibroblasts with breast cancer cells leads to a significant and steady increase in gene expression of SPP1, IGF1, POSTN and ACTA2. Since the proteins encoded by SPP1, IGF1 and POSTN are secreted from the cells making it technically challenging to develop a medium-high throughput assay with a readout of these proteins (i.e., this would require additional extraction, dilution and plating steps which would reduce the throughput of the assay). Therefore, we decided to develop the ICE assay using α-SMA an intracellular, structural protein. Moreover, it is the biomarker for myofibroblasts and activated CAFs. From the RT-qPCR experiments, we also concluded that the 7-day time point would be the optimum time point for the new assay.

### 3.3 Development of ICE assay

**FIGURE 4:**
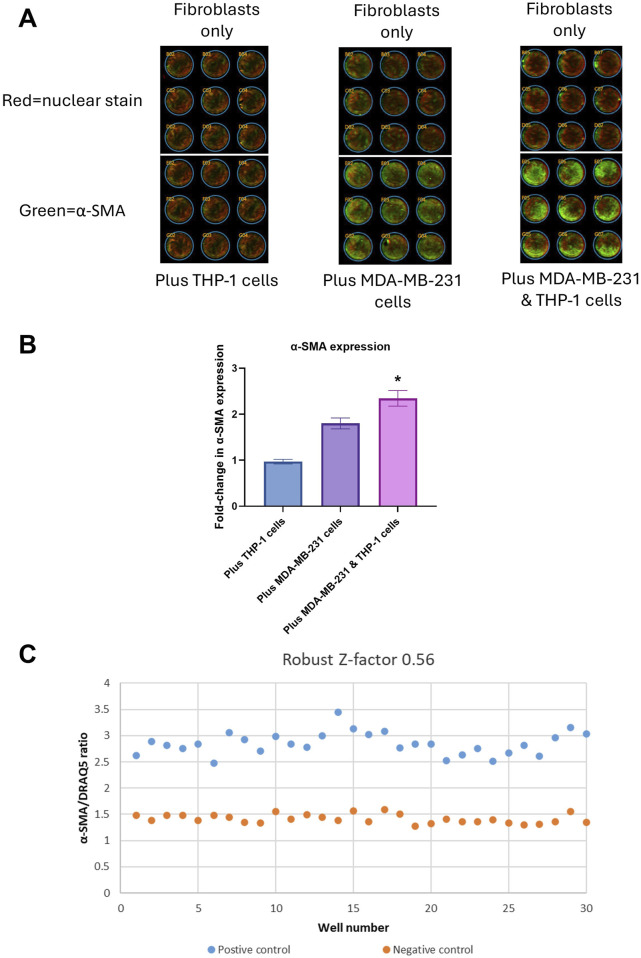
Assay development: **(A)** Infrared scanning image outputs showing fibroblasts without (top three rows) or with (bottom three rows) THP-1 cells only (left), MDA-MB-231 cells only (center) and MDA-MB-231 + THP-1 cells (right) using In-Cell ELISA (ICE) method. Green color denotes expression of α-SMA, red color denotes DRAQ5 nuclear staining. **(B)** Quantification of fold-change of α-SMA/DRAQ5 ratio. *p < 0.05 using Student’s t-test. **(C)** Robust Z′ value and a graph showing α-SMA/DRAQ5 ratios for each well of a 96-well plate. The data were obtained from three replicates from two donors.

After confirming that the cells we isolated from lung explants were fibroblasts and that they could be transformed to myofibroblasts using TGF-β1, we co-cultured these fibroblasts in the absence of TGF-β1 with and without MDA-MB-231 and THP-1 cells in 96 well plates for 7 days. At the end of 7 days incubation, the cells were fixed and stained for α-SMA. An increase in α-SMA expression would show an increase in myofibroblast numbers. The α-SMA expression was normalized to cell numbers by staining with DRAQ5 which was measured simultaneously with α-SMA staining as previously described (Ilg et al., 2022; Lapthorn et al., 2022). An example of staining obtained using this method is shown in Figure 4A. The top three rows in panel A show fibroblasts only and the bottom three rows show fibroblasts co-cultured with THP-1 cells (left), MDA-MB-231 cells (center) and MDA-MB-231 + THP-1 cells (right). Green denotes expression of α-SMA and red denotes nuclear staining. In Figure 4B, the same is shown when fibroblast were cultured with THP-1 cells only, MDA-MB-231 cells only and MDA-MB-231 cells + THP-1 cells. The measurement of α-SMA staining normalized to nuclear staining revealed that there was a significant 1.8-fold increase in α-SMA expression when lung fibroblasts were co-cultured with MDA-MB-231 cells (Figure 4B). When lung fibroblasts were co-cultured with MDA-MB-231 cells + THP-1 cells, the fold increase was significantly higher (2.3-fold; P < 0.05, Figure 4C). These results suggest that fibroblasts are activated when they are in contact with cancer cells. This activation is enhanced when monocytes are added. Since the highest fold change was achieved when three cell types were combined, we have decided to use this combination for the development of the 96 well plate assay.

An assay’s suitability for high throughput screening is measured using Z′ or RZ′ calculations. Z′ or RZ′ above 0.5 indicates that an assay is suitable for high throughput screening, meaning the fold change in the signal between negative and positive controls and the variation between repeats are within acceptable ranges. Z′ or RZ′ also include intra-assay variation in the calculations. In this study we used RZ′ instead of Z′, as it is less sensitive to outliers than Z′ and therefore would be more suitable for assay development using primary cells.

In order to achieve RZ′ above 0.5, we have optimized the ICE assay conditions by adjusting several culture conditions one at a time such as cell numbers, antibody concentrations and types of medium. The final optimized conditions are detailed in the Methods section. Using these conditions, we were able to achieve RZ′ of 0.56. A typical readout from one of the 96-well plates is shown in Figure 4C. Inter-assay variation was eliminated by including relevant negative and positive controls in each plate. Donor to donor variation was then 10%.

### 3.4 Secondary assay development

**FIGURE 5:**
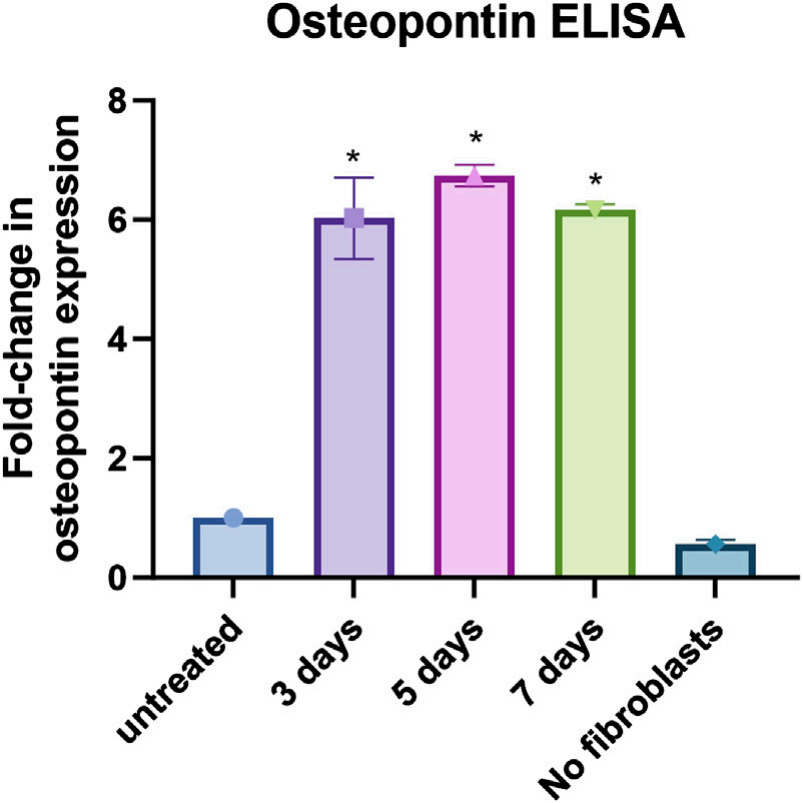
Osteopontin ELISA assay: Results from osteopontin ELISA assay for fibroblasts only (untreated), fibroblasts co-cultured with MDA-MB-231 and THP-1 cells for 3, 5 and 7 days, and MDA-MB-231 and THP-1 cells without fibroblasts (no fibroblasts). Data shown as fold-change in expression compared to fibroblasts only. *p < 0.05 using One-way ANOVA with multiple comparisons. The data were obtained from three replicates from two donors.

Typically, a battery of assays is used in phenotypic screening campaigns to build a chain of translatability (Moffat et al., 2017), which includes the use of secondary, functional screening assays. As SPP1, the gene for osteopontin, was the most upregulated gene we tested, and given its established role in metastasis formation, an osteopontin ELISA kit was utilized as a secondary assay. Co-culture of primary human lung fibroblasts with both MDA-MB-231 and THP-1 cells led to a significant increase in osteopontin secretion of around 6-fold compared to fibroblasts only (p < 0.0001) (Figure 5). The control samples of MDA-MB-231 and THP-1 without fibroblasts showed no significantly different secretion of osteopontin compared to fibroblasts only (p = 0.3306) (Figure 5). This makes the osteopontin ELISA a useful tool to confirm hits that were found when utilizing the primary screening assay.

## 4 Discussion

This study describes the development of a phenotypic screening assay designed to discover adjuvant treatments that could inhibit metastasis formation. Phenotypic drug discovery has been shown to produce more effective therapeutic leads compared to traditional target-based approaches (Swinney and Anthony, 2011; Moffat et al., 2014). Although phenotypic screening has proven successful in cancer therapy development (Moffat et al., 2014), using it to target the extracellular matrix (ECM) in metastatic niches is a novel strategy.

For lung fibroblasts, we used primary cells to enhance the translational relevance of our assay. We avoided using cell lines or immortalized cells since they are known to be inferior to primary cells in respect of their relevance to original tissue and preservation of cell-type specific functions (Pan et al., 2009). Since the lung tissues were anonymized, we did not have the genetic background of the donors therefore we are not able to comment on the effect of genetic background on the cell response. Nevertheless the difference between the donors in their cells’ response was not significant. In the future it would be interesting to develop a similar phenotypic screening assay using bone tissue-derived fibroblasts since bone is the second most common site for breast cancer metastasis.

For breast cancer cells, we used highly invasive MDA-MB-231 breast cancer cells, which often metastasize to the lung, where they would corrupt lung fibroblasts to form a metastatic niche (Jin et al., 2018). This cell line is derived from triple negative (negative for human epidermal growth factor receptor 2 [HER2], estrogen receptors [ER] and/or progesterone receptors [PR]) breast invasive ductal carcinoma with metastasis to the lungs (Lacroix and Leclercq, 2004). There are several breast cancer cell lines available to researchers; among which particularly MDA-MB231 and MDA-MB-435 cell lines have been shown to be highly metastatic to lungs (Lacroix and Leclercq, 2004). However, the provenance of MDA-MB-435 has been debated as it has been suggested that these cells might have originated from melanoma (Christgen and Lehmann, 2007). Bearing in mind that triple negative breast cancer accounts for 15%–20% of breast cancers albeit being the most difficult ones to treat (Yin et al., 2020), future research will be required to develop similar phenotypic screening assays using breast cancer cell lines which express HER2, ER and/or PR.

We aimed to mimic the metastatic microenvironment by co-culturing primary human lung fibroblasts with these breast cancer cells. The relevance of this co-culture is supported by its reliance on disease-relevant cell types rather than exogenous cytokine stimulation, further grounding the physiological fidelity of the system. For example, the driver for activation of CAFs by cancer cells may not only be TGF-β1 since other growth factors, cytokines and ligands have been suggested to be involved (Hu et al., 2022). This highlights strength of the phenotypic assay is to measure the phenotypic change regardless of the nature of the molecular driver. Furthermore, using α-SMA expression as a primary readout closely aligns the assay with clinical indicators of CAF activation and metastatic niche formation. Additionally, we achieved an RZ′ factor in the ICE assay above 0.5, indicating suitability for medium-high throughput screening.

Cancer microenvironment is not limited to cancer cells, CAF and immune cells; there are multitude of other cells which all play important roles in metastasis biology. A tissue culture or organoid system with all the cellular and molecular elements present would have been the ideal assay model (Nie et al., 2021). However, the throughput of an assay is inversely proportional to its complexity. Such an organoid assay would have a low throughput and therefore would be most suited as a secondary assay to test the hits from a high throughput primary assay.

We investigated the expression of selected 10 genes when fibroblasts were co-cultured with cancer cells for 3, 5 and 7 days. Since the gene for α-SMA (marker for CAF activation) was increased in 3 days and stabilized by 7 days, we decided to use 7 days time point as the end of the assay. It would be interesting to investigate the phenotypic changes that occur beyond this time point in future studies.

In the co-culture of cancer cells with fibroblasts osteopontin (SPP1) emerged as the most upregulated gene. Its relevance in metastasis formation has been described in detail (Anborgh et al., 2010; Shevde et al., 2010; Jia et al., 2016). Elevated osteopontin levels relate to poor patient prognosis (Kumari et al., 2024), and both secretory (Li et al., 2013) and intracellular osteopontin (Jia et al., 2016) have been shown to induce mesenchymal–epithelial transition (MET) to facilitate the formation of metastases. Moreover, osteopontin deficiency has been shown to reduce metastasis formation in experimental models (Moorman et al., 2020). We utilized an ELISA-based secondary assay to validate osteopontin levels at the protein level, addressing the limitation that mRNA expression does not always correlate with protein expression (Schwanhäusser et al., 2011). This two-step assay approach provides a scalable platform for screening compounds that could prevent fibroblast activation and metastatic niche formation.

Osteopontin is known to be released by THP-1 cells (Bai et al., 2020)and SPP1 expression is reported in macrophages in breast cancer (Zhao et al., 2024). It can therefore be deduced that some of the osteopontin measured in our secondary assay might be released from THP-1 cells. However, when THP-1 cells were co-cultured with cancer cells, no significant osteopontin was measured (Figure 5) suggesting that fibroblast + monocyte + cancer cell triple culture is required for significant osteopontin release. Interestingly, a similar condition is required for α-SMA expression: only in the presence of all three cell types, a significant increase in α-SMA expression is observed (Figure 4). It can be hypothesized from these observations that fibroblast + monocyte + cancer cell combination is a prerequisite for the release of osteopontin which in turn regulates CAF activation. Further research would be required to test this hypothesis.

Several additional key genes associated with metastasis and CAF function were differentially regulated. ACTA2, the gene for the myofibroblast marker α-SMA shows significant differential regulation, which is in line with reports of CAFs expressing the protein (Öhlund et al., 2017; Bartoschek et al., 2018; Serini et al., 1998). Tenascin C has been described to increase lung metastasis (Sun et al., 2019) and to promote the survival of breast cancer cells forming pulmonary micrometastases (Oskarsson et al., 2011). Periostin, which interacts directly with other ECM molecules such as collagen I, tenascin C or fibronectin, has been described to promote metastasis (Xu et al., 2022). IGF-1 has been described to facilitate tumorigenesis and metastatic spread in other cancers (Liu et al., 2023).

RB transcriptional corepressor like 1 (RBL1), which plays a role in regulating cell cycle progression, differentiation, and gene expression, was downregulated after 7 days of co-culture. This is not surprising since this gene has been suggested to be involved in the development and progression of various cancers, its loss has been associated with poor outcomes in multiple cancer types (Huang et al., 2024). TGFBR3 was downregulated in our experiments which is also in line with previous reports which suggest that TGFBR3 is a tumor suppressor (Dong et al., 2007). Although Smad4 and thrombospondin have been suggested to be involved in cancer metastasis (Kaur et al., 2021; Tan et al., 2021), their expression did not show a significant change in our experiments.

We used THP-1 cells as a source of monocytes to mimic the immune cell component of the cancer metastasis microenvironment. THP-1 cells are non-differentiated monocytes and can be differentiated to macrophages using phorbol 12-myristate 13-acetate (PMA) and then further polarized to M1 macrophages using interferon gamma or M2 macrophages using lipopolysaccharide (Genin et al., 2015). Future research will be required to investigate how M1/M2 macrophages can be incorporated into phenotypic assays similar to the one developed in this study.

In qPCR experiments, the size of error bars for relative expression changes increases significantly when both the minimum and maximum mRNA copy numbers are considered, rather than just the average. This is because error propagation, a critical aspect of gene expression analysis, accounts for variability at every step, including RNA extraction, reverse transcription, and amplification efficiency. The relative expression is typically calculated using a ratio of target to reference gene expression, and errors from both measurements compound when propagating through calculations. When reporting minimum and maximum copy numbers, the range of possible relative expression values broadens, amplifying the uncertainty. To ensure the reliability and reproducibility of qPCR data, we have calculated and presented 95% confidence intervals (CI) alongside the data, thus providing a statistical measure of variability and enhances the interpretability of results.

Overall our study establishes a novel phenotypic assay for screening anti-metastatic adjuvants in a model that mirrors the lung microenvironment. With further optimization, including adaptation to higher-density formats (e.g., 384- or 1536-well plates), this system could support large-scale screens, offering a promising tool for identifying agents that specifically inhibit CAF-mediated ECM remodeling without inducing tumor dissemination.

### 4.1 Limitations

This study is limited by the fact that not all of the major ECM players or markers of cellular fibrosis have been tested in our primary or secondary assays. Future research and assay development would be required to investigate whether other markers such as E-cadherin (Chan et al., 2015) are up- or downregulated in our assay system. The choice of protein for our secondary assay is currently osteopontin, but with further research and assay development this choice may be changed, or it may be necessary to add further biomarkers as secondary assays.

Another limitation relates to the inability to locate gene expression changes to specific cell types (fibroblasts or cancer cells) in the co-culture, since RNA isolation was performed on the bulk cell population. Cell-specific resolution would require fluorescence-activated cell sorting (FACS) or similar methods prior to RNA extraction, an approach that was outside this study’s scope.

Another limitation is the lung-specific focus of this assay. Although lung fibroblasts are biologically relevant for modeling breast cancer metastasis to the lung, this approach may limit the ability to generalize the relevance of identified compounds to other common metastatic sites, such as bone or brain. This limitation reflects the heterogeneity observed across CAF populations, which may drive site-specific metastatic growth (Bartoschek et al., 2018). Nevertheless, targeting the metastasis initiating bottleneck after surgical debulking and systemic adjuvant treatment presents a promising strategy to prevent formation of a metastatic niche and might hold promise for therapy. Elucidating what drives the tropism of cancer metastasis and finding ways to target all metastatic spread remains a challenge.

We have identified some genes which were significantly upregulated when lung fibroblasts and breast cancer cells were co-cultured. Among them, the genes for α-SMA and osteopontin showed the highest upregulation which were then taken forward for the development of the ICE assay where lung fibroblasts and breast cancer cells were co-cultured with monocytes. We did not measure the gene and protein expression of biomarkers other than osteopontin and α-SMA when all three cell types were cultured together. Future research would be required to investigate whether other biomarkers are differentially up- or downregulated when all three cell types are cultured together.

### 4.2 Future directions

This co-culture assay which uses lung fibroblasts, cancer cells and monocytes can now be further developed into a medium-high throughput phenotypic screening assay. Hits from the primary screen can be further triaged using the secondary osteopontin assay.

### 4.3 Conclusions

This study provides proof of concept that a co-culture of fibroblasts, monocytes and cancer cells can be developed in a 96 well plate format that can measure activation of CAFs. This assay and the accompanying secondary osteopontin assay have the potential to identify novel adjuvant drugs to prevent metastasis.

## Data Availability

The original contributions presented in the study are included in the article/Supplementary Material, further inquiries can be directed to the corresponding author.
